# Infarction of a polyp within a mesenteric cyst: An unusual presentation as an acute abdomen

**DOI:** 10.4103/0971-9261.70647

**Published:** 2010

**Authors:** Sonia Gon, Bipasa Majumdar, Aditi Bhattacharyya, Tushar K. Das, Indranil Chatterjee

**Affiliations:** Department of Pathology, R G Kar Medical College & Hospital, Kolkata, India; 1 Department of Pediatric Surgery, R G Kar Medical College & Hospital, Kolkata, India

**Keywords:** Acute abdomen, infarcted polyp, mesenteric cyst

## Abstract

A case of mesenteric cyst in a five-year-old male child who presented with acute abdomen due to an infarcted polyp present within the cyst is reported. To the best of our knowledge, such an event has never been reported in the literature previously.

## INTRODUCTION

Mesenteric cysts are rare, intra-abdominal lesions arising with an incidence of 1/20,000 in children and 1/100,000 in adults.[1] They are usually asymptomatic and incidentally detected during physical or radiological examination or when a complication like hemorrhage and rupture due to blunt trauma, volvulus of the cyst, intestinal obstruction, abscess formation or malignant transformation of the cyst develops.[2]

## CASE REPORT

A five-year-old male child was admitted with acute pain abdomen and vomiting of two days duration. The pain was severe, sudden in onset, non-radiating and was diffuse. The abdominal examination revealed tenderness in the abdomen with no rigidity, slight guarding, and no palpable mass. His pulse and respiratory rates were 120/min and 32/min, respectively. Routine hematological examination was within normal limits except for mild leucocytosis and slightly raised C-reactive protein. An ultrasonography (USG) of abdomen revealed a cystic lesion in relation to the small intestine mesentery. Computed tomogram (CT) scan showed evidence of intraperitoneal multiseptate cystic lesion measuring 7 cm × 4.10 cm, located at the region of small bowel mesentery and displacing the adjacent gut loops, predominantly consisting of water attenuation area interspersed with some septa, favoring the possibility of mesenteric cyst.

At laparotomy, a thin-walled dumbbell-shaped mesenteric cyst was seen attached to the ileal mesentery 15 cms proximal to the ileocecal junction. The cyst was not communicating with the intestinal lumen and did not share the wall but had a common vascular supply. Surgical resection of the cyst along with a small part of the ileum was done followed by end-to-end anastomosis to restore the intestinal continuity. Postoperative period was uneventful and the patient was discharged on the sixth post-operative day.

### Histopathology

**Figure 1:**
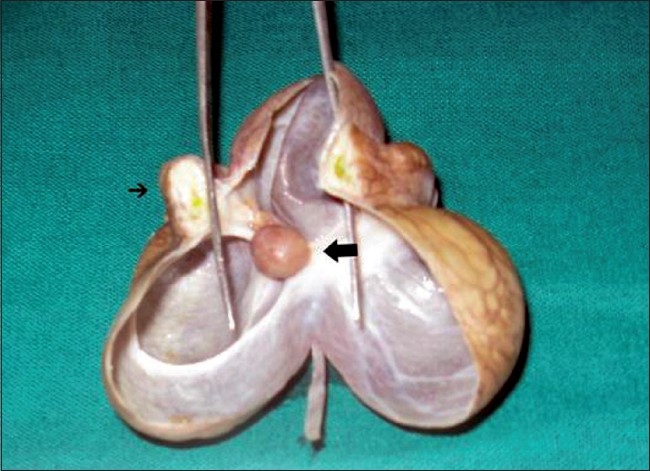
Gross photograph showing thin-walled, multiloculated cyst with a small polyp (thick arrow) measuring 1.4 cm in its greatest dimensions attached to the inner surface of the cyst wall through a small stalk and part of the small intestine (thin arrow).

Grossly, the cyst was thin-walled, contained pale straw-colored fluid and was multiloculated. It measured 7.1 cm in its greatest dimensions. Cut surface revealed a small polyp measuring 1.4 cm in its greatest dimensions attached to the inner surface of the cyst wall through a small stalk. The cut surface of the polyp was grayish brown and congested [Figure 1]. Microscopically, the cyst wall was composed of fibro-collagenous tissue lined by flattened to low cuboidal epithelial cells. The attached polyp was completely infarcted and showed congested blood vessels, a few hemosiderin laden macrophages and plenty of inflammatory cells near the stalk. Since the polyp was completely infarcted, exact morphological categorization could not be done.

## DISCUSSION

Mesenteric cysts are rare benign tumors that can be classified histologically as lymphatic, mesothelial, enteric, urogenital, dermoid cystic and pseudo-cystic origin.[3] They can be localized anywhere along the gastrointestinal mesentery with 66% found in the small intestine mesentery and 33% in the mesentery of the large intestine, usually in the right colon.[2,4]

Mesenteric cysts commonly occur as single lesion, but multiple lesions have also been reported. They can be unilocular or multilocular and may contain serous, chylous, hemorrhagic, or infective fluid. Cyst contents are sometimes related to their etiologic derivation. Cysts that develop after occult trauma may contain hemorrhagic content; chylous fluid may be found in jejunal cysts and in cysts that are intimately involved in the lymphatic pathway; and serous fluid is typically encountered in cysts of the ileum and colonic mesentery.[5] The present case also had a multiloculated cyst with serous contents and an ileal mesenteric location.

The symptoms are variable, non-specific and include pain (82%), nausea and vomiting (45%), constipation (27%) or diarrhea (6%). An abdominal mass may be palpable in approximately 61% of patients.[6] It may also present with acute symptoms secondary to complications such as obstruction (volvulus, extrinsic compression or entrapment in pelvis), rupture, and hemorrhage into cyst, infection or abscess formation.[2] The most common acute presentation in children is small bowel obstruction with a possible volvulus and an associated intestinal infarction.[7] However, infarction of a polyp within a mesenteric cyst leading to an acute presentation as found in the present case has never been reported in the literature.

In suspected cases, the diagnosis should be confirmed with USG and CT scan.[8,9] The treatment of choice is a complete surgical excision. Resection of the adjacent gut may be required due to the involvement of the mesenteric vessels. The advent of laparoscopic surgery has allowed resection of these cysts to be achieved without full laparotomy.[10]

To conclude, even though, mesenteric cysts are rare and usually lack symptoms, they must be kept in mind in cases of non-specific abdominal symptoms. Preoperative imaging usually provides the diagnosis, and surgical excision is the treatment of choice with excellent results. However, histopathology is the main stay for the diagnosis and may reveal an unusual pathology related to its acute presentation.
